# Different Colours, Different Outcomes: Tank Colour Shapes Larval Survival, Growth, and Endocrine Response in *Cichlasoma dimerus*

**DOI:** 10.3390/ani16030466

**Published:** 2026-02-02

**Authors:** Agustina C. Beriotto, María P. Di Yorio, Julieta E. Sallemi, Carlos A. Alvarez-González, Paula G. Vissio

**Affiliations:** 1 Departamento de Biodiversidad y Biología Experimental, Facultad de Ciencias Exactas y Naturales, Universidad de Buenos Aires, Buenos Aires 1428, Argentina; agusberiotto93@gmail.com (A.C.B.); mariapauladiyorio@gmail.com (M.P.D.Y.); julietasallemi@gmail.com (J.E.S.); 2 Instituto de Biodiversidad y Biología Experimental y Aplicada (IBBEA), Universidad de Buenos Aires-CONICET, Buenos Aires 1428, Argentina; 3 Laboratorio de Fisiología en Recursos Acuáticos (LAFIRA), División Académica de Ciencias Biológicas, Universidad Juárez Autónoma de Tabasco, Carretera Villahermosa-Cárdenas Km 0.5, Villahermosa 86039, Tabasco, Mexico

**Keywords:** development, fish, larvae, aquaculture, skin pigmentation, sex proportion, skeletogenesis

## Abstract

Early environmental conditions play a critical role in shaping fish physiology and behaviour, with tank colour emerging as a relevant yet often overlooked factor in aquaculture and experimental research. This study investigated how rearing tank colour affects larval performance in the cichlid fish *Cichlasoma dimerus*. Larvae were reared in white, light-blue, or grey tanks, and survival, growth, pigmentation, sex ratio, skeletal development, and the endocrine responses of somatolactin (Sl) and growth hormone (Gh) were evaluated. Survival was significantly lower in white tanks. Conversely, larvae reared in white tanks reached a higher final body weight, while total length showed a similar but non-significant trend. Gh-immunoreactive cells exhibited significantly larger nuclear areas in larvae reared in white tanks and a tendency toward smaller cytoplasmic areas compared with those from light-blue tanks. Melanophore number did not differ among treatments, whereas larvae from grey tanks showed a higher number of Sl-immunoreactive cells. Sex ratios tended to be female-biased in white tanks. Skeletal development did not differ among tank colours and followed the expected chondrogenesis and ossification sequence. Overall, tank colour influenced multiple aspects of larval development, particularly survival, growth, and endocrine responses, underscoring its relevance in fish rearing and experimental design.

## Simple Summary

Conditions experienced early in life can strongly influence how fish grow, survive, and develop. One factor that is often overlooked in fish rearing is tank colour. In this study, we examined how tank colour affects the development of larvae of the cichlid fish *Cichlasoma dimerus*. Larvae were reared in white, light-blue, or grey tanks, and we evaluated survival, growth, pigmentation, skeletal development, sex ratio, and hormone-related responses. We found that larvae reared in white tanks had lower survival but reached a higher body weight compared with those reared in the other colours. Tank colour also affected hormone-related traits linked to growth and skin pigmentation, although skin pigmentation itself did not differ among treatments. Skeletal development followed the expected pattern across all colours, and a higher proportion of females was observed in white tanks. Overall, these results demonstrate that tank colour influences multiple aspects of fish development and should be considered when optimizing early-life rearing conditions, fish welfare, and experimental design.

## 1. Introduction

Environmental conditions to which larvae are exposed during development play a crucial role in shaping fish physiology, morphology, and behaviour [1,2,3]. Among these factors, tank colour has emerged as a relevant yet often overlooked variable in both aquaculture and experimental research [4]. Tank colour is of interest from both theoretical and practical perspectives, especially given how easy it is to manufacture tanks in a wide range of colours. Nevertheless, most commercial facilities employ a limited palette [3,4].

The effects of tank colour on fish welfare are complex and species- and developmental stage-specific (e.g., embryos, larvae, or juveniles). In some cases, colour preferences appear to be ecologically matched to the species, but this is not always the case. Additionally, colour preferences cannot be generalized across families. Consequently, the most suitable colour for each species is usually determined through trial and error [4]. For example, white tanks promote growth in *Liza ramada*, reduce growth in *Oncorhynchus mykiss*, and have no effect on *Cyprinus carpio* [5,6,7]. Several studies indicate that tank colour can also influence skin pigmentation, stress responses, survival, and behaviour. For example, white tanks generally lighten fish skin pigmentation and reduce hypermelanosis, whereas black tanks tend to darken the animals [4,8,9]. Tank colour can also have an effect on stress, with certain colours associated with elevated cortisol or glucose levels, which, when observed during critical periods of sex determination, can lead to biased sex ratios [10,11]. Behavioural effects have also been reported; for instance, in *Latris lineata* larvae, red tanks increased “walling behaviour”, resulting in a higher incidence of jaw deformities [12].

*Cichlasoma dimerus* (Heckel, 1840) is a Neotropical cichlid belonging to the family Cichlidae, the most species-rich non-Ostariophysan family among freshwater fishes worldwide [13]. It inhabits both lentic and lotic environments across the Paraná, Paraguay, and Uruguay river basins in South America [14]. This species has become an excellent model for studies on neuroendocrinology, development, behaviour, toxicology, and growth [15,16,17,18,19,20,21,22,23,24,25,26,27,28]. *Cichlasoma dimerus* readily adapts to captivity, tolerates a wide range of temperatures, and reproduces efficiently under controlled laboratory conditions. Spawning occurs on substrates with external fertilization and biparental care, yielding large spawns that facilitate larval studies [29].

Importantly, previous studies have demonstrated that *C. dimerus* exhibits a pronounced plastic response to tank background colour (black vs. white) at different life stages. Adults maintained in black tanks exhibited a higher melanophore number in scales, a higher number of somatolactin (Sl)-immunoreactive (Sl-ir) cells, larger Sl-ir cell areas, and increased expression of *sl* and its receptor compared to those maintained in white tanks [30,31]. Somatolactin is a pituitary hormone involved in multiple processes, including background adaptation and the regulation of pigment movements [32]. Both growth hormone (Gh) and Sl appear to be modulated by melanin-concentrating hormone (Mch), linking pigmentation and growth to environmental cues such as tank colour in this species [33,34]. Consistently, juveniles kept in white tanks showed higher *mch* expression and greater growth in length and weight [34]. In larvae, Sl-ir cell numbers and head melanophore counts were also increased in fish reared in black tanks compared to those reared in white ones [35]. Together, these findings highlight the suitability of *C. dimerus* as a model to investigate how visual environmental cues shape endocrine regulation, growth, and skin pigmentation.

Although previous studies have focused mainly on extreme colour contrasts (black vs. white), it remains unclear how more moderate, aquaculture-relevant tank backgrounds influence larval development. Therefore, the aim of this study was to investigate how a subtler range of tank colours, particularly during early ontogeny, affects larval performance in *C. dimerus*, including survival, somatic growth, pigmentation, sex ratio, skeletal development, and the endocrine responses of Sl and Gh.

## 2. Materials and Methods

### 2.1. Experimental Tanks

**Figure 1:**
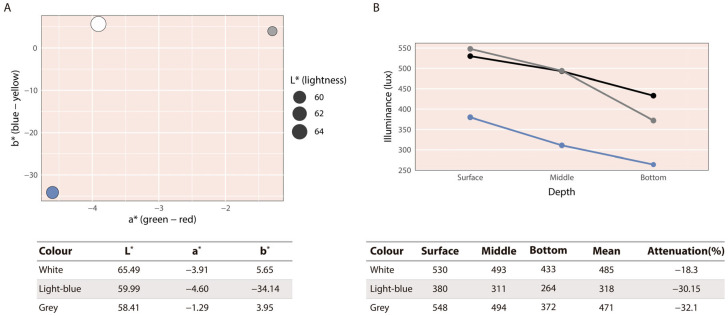
(**A**) CIELab values (L*, a*, b*) for the experimental tanks. (**B**) Illuminance (lux) at surface, middle, and bottom, with mean values and percentage light attenuation for each tank colour. Note that the white tank is represented by a black line.

Photographs of the three tanks with water were taken, which were all placed on the same shelf where the experiment was conducted to ensure the same lighting conditions. A black sheet was placed inside each tank, and a black balance was performed to standardize image exposure. The images were then processed in R, where values in the CIELab colour space were obtained, including lightness (L*) and the chromatic components a* (green–red) and b* (blue–yellow), to quantitatively characterize colour differences among treatments. Global colour differences among tanks were subsequently calculated using ΔE_00_ (CIEDE2000). All calculations were performed in R version 4.4.1 using the compare_coulour function from the farver package. Regarding the CIELab parameters, L* was highest in white tanks, whereas both light-blue and grey tanks showed comparably lower L* values. Along the a* axis, the grey tank had values close to zero, whereas white and light-blue tanks displayed negative values, indicating a shift towards green. On the b* axis, white and grey tanks showed slightly positive values, while the light-blue tank exhibited a markedly negative value, reflecting a stronger blue component (Figure 1A). Global colour differences among tanks were greatest between light-blue and white tanks (ΔE_00_ ≈ 26.6), followed by light-blue and grey (ΔE_00_ ≈ 24.5), whereas white and grey tanks were more similar (ΔE_00_ ≈ 7.0).

Additionally, light intensity in each tank colour was measured using a lux meter (Digital luxometer TES-1330; Tes Electrical Electronic Corp., Taipei, Taiwan) to quantify differences in illuminance among tank colours. Lux values were recorded at three depths (surface, middle, and bottom), and average light intensity and percentage attenuation per tank were calculated. Illuminance was highest in white tanks, slightly lower in grey tanks, and lowest in light-blue tanks. In all cases, light intensity decreased with depth, but attenuation was greater in grey and light-blue tanks compared to white. The decline between the middle and bottom levels was markedly steeper in grey tanks, indicating greater light attenuation under this background colour (Figure 1B).

All colour and illuminance measurements were performed at the beginning of the experiment; this occurred once the tanks were fully set up and before introducing the eggs.

### 2.2. Rearing Conditions

Fertilized eggs were obtained from three independent spawnings, each derived from a different pair of wild-caught reproductive adults of *C. dimerus*. These adults were captured in the Esteros del Riachuelo, Corrientes, Argentina, and subsequently transferred to the laboratory, where they were acclimated and maintained in 400 L freshwater glass aquaria under a 12:12 h light/dark cycle at 25 ± 2 °C. Fish were fed once daily with commercial pellets (Awamix Iniciador, #310; Mixes del Sur, Buenos Aires, Argentina; approximate composition: 48% protein, 4% fiber, 14% lipids, 2.5% calcium, 1.25% phosphorus).

Each of the three spawnings was divided into three groups and randomly assigned to the three experimental tank colours -white, light-blue, and grey- so that each spawning was represented in all three colour conditions. This design was replicated three times, using a different pair of reproductive adults for each spawning, resulting in a total of nine experimental tanks. Tanks were rectangular (40 cm length × 20 cm width × 30 cm height), with a total volume of 17 L. Each tank had between 440 and 480 fertilized eggs.

Larvae were maintained until the end of the experiment, at 90 days post-hatching (dph), under the same photoperiod and temperature conditions as the adults. Tanks were syphoned daily, and 30% of the water volume was replaced with fresh dechlorinated water. From 5–6 to 12 dph, larvae were fed with *Artemia* nauplii (Sanders Co., South Ogden, UT, USA). Between 12 and 20 dph, a mixed diet of *Artemia* nauplii and ground commercial pellets (Awamix Iniciador, #703; Mixes del Sur, Buenos Aires, Argentina; approximate composition: 49% protein, 4% fiber, 15% lipids, 2.5% calcium, 1.25% phosphorus) was provided. From 20 to 90 dph, larvae received only commercial pellets. In all cases, feeding was carried out ad libitum, four times per day.

Animals were handled according to the Principles of Laboratory Animal Care (Guidelines on the care and the use of fish in research, teaching and testing, Canadian Council on Animal Care, 2005) approved by the Comisión Institucional para el Cuidado y Uso de Animales de Laboratorio, Facultad de Ciencias Exactas y Naturales, Universidad de Buenos Aires, Argentina (CICUAL, Protocol No. 231; approval date: 12 May 2025).

### 2.3. Survival Rate

Throughout the experiment, mortality was recorded, and dead individuals were removed from each tank. These data were used to calculate the survival rate at the end of the study and to monitor tank density, which was maintained constant across colour treatments by adjusting the water volume according to the number of larvae. Survival analyses were conducted using the survival package in R [36,37], which enables the generation of Kaplan–Meier survival curves and their subsequent comparison using log-rank tests.

### 2.4. Skin Pigmentation

At 5, 12, and 25 dph, 5 larvae per tank colour and per replicate (*n* = 15, N = 45) were collected, anesthetized in ice-cold water, and submerged in a 20 µg mL^−1^ dopamine solution to induce melanophore aggregation. Larval heads were photographed under a light microscope while maintained at 0 °C to reduce movement [35]. Melanophores were quantified in a defined dorsal head region extending approximately from the midpoint of the eyes to the posterior part of the head. This region was consistently recognized by its distinct orange skin pigmentation. Melanophores were counted using the Cell Counter plugin in ImageJ version 1.53a, and the corresponding anatomical area was also measured for each larva.

Data followed a Poisson distribution and were analyzed using the glmer.nb function from the lme4 package [38] in R version 4.4.1 [37]. A negative binomial model was used to correct for overdispersion, and an offset term was included to account for differences in the measured morphological area. Group-level dependencies (replicate and tank) were included as random effects. Post hoc contrasts were performed using the emmeans package.

### 2.5. Somatic Growth

Somatic growth was assessed by measuring total length and body weight at 5, 12, 25, 60, and 90 dph. At each time point, 10 larvae per tank per replicate were measured, except at 60 dph, when only 5 larvae per tank were used (*n* = 45, N = 135). Before measurement, larvae were anesthetized in ice-cold water. At 5 and 12 dph, due to limitations of the balance, weight was recorded from pooled samples of larvae per treatment. For total length measurements, larvae were photographed at each time point and measured individually using ImageJ version 1.53a.

Data were normally distributed and analyzed using the gls function from the nlme package in R version 4.4.1 [37,38]. Although larval density was controlled throughout the experiment, it was initially incorporated into the model to assess potential interactions with tank colour and its possible influence on somatic growth. Group-level dependencies (i.e., replicate and tank) were accounted for in the correlation structure of the model. Model assumptions of normality and homoscedasticity were evaluated, and violations of homoscedasticity were addressed using the varIdent variance structure, with replicate as a grouping factor, which provided the best model fit. Post hoc contrasts were conducted using the predictmeans package. As the same larvae were measured for both total length and weight, a Bonferroni correction was applied, setting the new significance threshold at *p* = 0.025.

### 2.6. Sl and Gh Response

To assess Sl and Gh responses, immunohistochemical analyses were performed at 60 dph for Sl and 90 dph for Gh. In both cases, 5 larvae per tank colour per replicate (*n* = 15, N = 45) were fixed in Bouin’s solution for 24 h, dehydrated through a graded ethanol series, and embedded in Paraplast^®^ (Leica Biosystems, Nussloch, Germany). Samples were coronally sectioned at 7 μm and mounted on gelatin-coated slides. Sections containing the pituitary gland were deparaffinised in xylene, rehydrated through a descending ethanol series, and rinsed in phosphate-buffered saline (PBS; pH 7.4). Immunohistochemical procedures followed those previously described by Di Yorio et al. [16,39].

Briefly, after rehydration, sections were incubated for 10 min in 0.3% (*v*/*v*) hydrogen peroxide to block endogenous peroxidase activity, rinsed in PBS, and incubated for 1 h at room temperature (RT) in a blocking solution containing 5% (*w*/*v*) non-fat dry milk in PBS. Slides were then incubated overnight at 4 °C with either rabbit anti-*Sparus aurata* Sl antiserum (1:1500; [40]) or anti-*Odontesthes bonariensis* Gh antiserum (1:1500; [41]). After washing with PBS, sections were incubated for 1 h at RT with biotinylated anti-rabbit IgG (1:500 in PBS; Sigma-Aldrich, Burlington, MA, USA), followed by 1 h incubation with peroxidase-conjugated streptavidin (1:500 in PBS; Invitrogen, Carlsbad, CA, USA). Immunoreactive products were visualized using a DAB Substrate Kit (Cell Marque, Rocklin, CA, USA). Sections were counterstained with haematoxylin, mounted with synthetic Canada balsam (Biopack), and examined under an Olympus CH2 microscope (Olympus, Tokyo, Japan) equipped with a digital camera (Hayear, model HY-5300, Shenzhen, China). Antibody specificity had been validated previously [42].

Quantification of Sl-immunoreactive (Sl-ir) cells was performed on the section containing the highest number of immunoreactive cells for each pituitary. Data followed a Poisson distribution and were analyzed using the glmer.nb function from the lme4 package [38] in R version 4.4.1 [37]. A negative binomial model was used to account for overdispersion. Replicate and tank were included as random effects. Post hoc contrasts were performed using the emmeans package.

For Gh, photographs were taken from two separate areas within the proximal pars distalis, which displayed a distinguishable spatial distribution of Gh–ir cells among larvae. The two areas were separated by at least four sections (approximately 24 µm) to avoid counting the same cells more than once. Only cells with clearly visible nuclei were measured. Nuclear and total cell areas were quantified, and the cytoplasmic area was calculated by subtraction. Separate analyses were performed for nuclear and cytoplasmic areas. Data followed a normal distribution and were analyzed using the gls function from the nlme package in R version 4.4.1 [37]. Because cells within larvae and larvae within tanks are not independent observations, a compound symmetric correlation structure (corCompSymm) was applied, with individual cells nested within larvae, larvae nested within tanks, and tanks nested within replicates. Heteroscedasticity was modelled using the VarPower function. The interaction between region and tank colour was evaluated and cell area was included as a covariate. Post hoc contrasts were conducted using the emmeans package. As the same individuals were used for both cytoplasmic and nuclear measurements, Bonferroni correction was applied, setting the significance threshold at *p* = 0.025.

### 2.7. Sex Ratio

At the end of the experiment, 10 larvae per tank colour per replicate (*n* = 30, N = 90) were fixed in Bouin’s solution for 24 h, dehydrated through a graded ethanol series, and embedded in Paraplast^®^ (Leica Biosystems, Nussloch, Germany). Samples were coronally sectioned at 10 μm and mounted on gelatin-coated slides. Sections containing gonads were deparaffinised with xylene, rehydrated through a descending ethanol series, and rinsed in phosphate-buffered saline (PBS; pH 7.4). Gonads were stained with haematoxylin and eosin (H&E) for histological sex identification.

Sex ratio data followed a Bernoulli distribution and were analyzed using the glmer function from the lme4 package [38]. Dependencies within groups, such as replicate and tank, were accounted for by introducing them as random effects.

### 2.8. Skeletal Development

At 5, 12, and 25 dph, 10 larvae per tank colour per replicate (*n* = 90, N = 270) were collected, anesthetized in ice-cold water, and fixed in 4% buffered formaldehyde (pH = 7.4) at 4 °C for 24 h. Samples were then dehydrated through a graded ethanol series (25%, 50%, 75% and 100%) for 30 min at each concentration. Skeletal staining was performed following a modified protocol based on Beriotto et al. (2023) [43]. Briefly, eyes were removed to improve visualization of cranial cartilage and bone structures. Larvae were rinsed twice in distilled water (5 min each), then incubated overnight in an alcian blue 8GX solution (Mallinckrodt) containing 70% ethanol and 50 mmol L^−1^ MgCl_2_. Samples were rehydrated through decreasing ethanol concentrations (70%, 40%, and 15%, 30 min each, with a midpoint renewal), followed by a final wash in distilled water. To remove pigmentation, larvae were bleached in a solution of one volume of 10% H_2_O_2_ and nine volumes of 1% KOH for approximately 1 h. After bleaching, they were washed in 1% KOH for 5 min and then incubated in an alizarin red solution (0.007% in 1% KOH; Biopack, C.I. 58005, Buenos Aires, Argentina) for 2.5 h. Samples were washed again in distilled water and then in 1% KOH (5 min each). Finally, larvae were cleared and preserved by transferring them through a graded glycerol series (40% glycerol in 1% KOH, 70% glycerol in 1% KOH, 20 min each) and stored in 100% glycerol. Based on previous studies of *C. dimerus* skeletogenesis [43], the double staining technique was only applied to 5 and 12 dph larvae. For 25 dph larvae, alizarin red was used instead, as little to no difference in cartilage structures was observed.

All the larvae were analyzed using an optical stereomicroscope. Cartilage structures were categorized as either absent, present, or showing the onset of cartilage replacement. Bone structures were categorized as either absent, showing the onset of ossification (approximately 5%), partial ossification (less than 50%), advanced ossification (more than 50%), or fully ossified (100%). These categorical data were organized into individual-by-structure matrices and transformed into numerical values for multivariate analyses. Structures with zero variance or those containing missing values were excluded. In a few cases, missing values were imputed using the column mean within the corresponding treatment group to avoid excluding individuals with incomplete staining. Matrices were scaled before analysis, and Principal Component Analysis (PCA) was performed using the prcomp function in R (version 4.4.1) [37]. To test for differences in overall skeletal development patterns among tank colours, PERMANOVAs were conducted using the adonis2 function from the vegan package [44]. The variance explained by each principal component was calculated, and the relative contribution of each structure to PC1 and PC2 was estimated.

## 3. Results

### 3.1. Survival Rate

**Figure 2:**
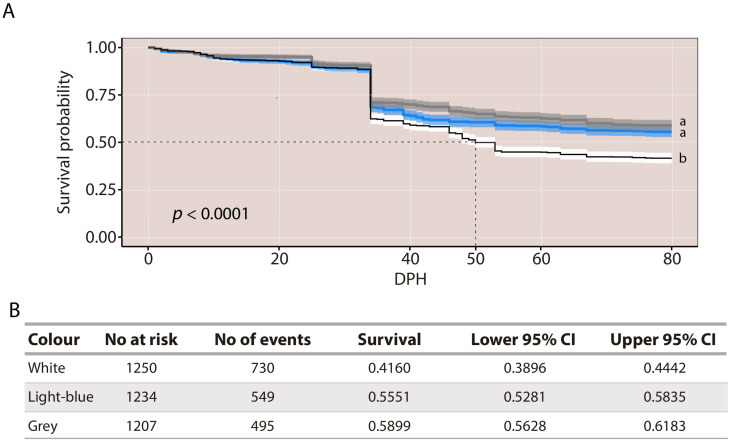
(**A**) Kaplan–Meier survival curves with 95% confidence intervals for larvae reared in the three tank colours. The dashed line indicates the median survival time for larvae reared in white tanks. (**B**) Survival rate with 95% confidence intervals at the end of the three experiments. No at risk represents the number of larvae at the beginning of the experiment, and No of events represents the number of deaths throughout the experiment. Different letters indicate statistically significant differences among groups; groups sharing the same letter are not significantly different.

Overall, survival curves across the three tank colours exhibited similar trends; however, when mortality events occurred, they were more pronounced in larvae reared in white tanks (Figure 2A). Consistently, by the end of the experiment, the survival rate of larvae reared in white tanks (41.6%) was significantly lower than that of those reared in light-blue (55.5%) and grey tanks (58.99%) (log-rank test: χ^2^ = 74.2, df = 2, *p* < 0.0001; Figure 2A,B). No significant differences were observed between the light-blue and grey tank treatments. A marked decline in survival was observed at approximately 30 dph in a single experimental replicate, affecting all tank colours simultaneously. This drop corresponded to an isolated event inherent to the rearing conditions rather than to an underlying biological process. Notably, the only instance in which survival dropped below 50% occurred in larvae reared in white tanks, around 50 dph (Figure 2A).

### 3.2. Skin Pigmentation

**Figure 3:**
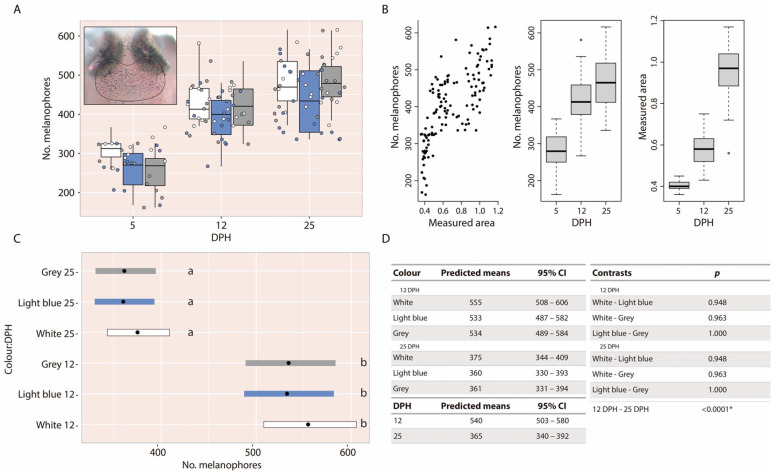
(**A**) Photograph of a 12 dph larva showing the measured dorsal head area, alongside a boxplot illustrating the number of melanophores for the three tank colours at 5, 12, and 25 dph. (**B**) Correlations between the number of melanophores, dph, and the measured area. (**C**,**D**) Contrasts, predicted means, and 95% confidence intervals for the number of melanophores per mm^2^ for larvae reared in the three tank colours at 12 and 25 dph. Different letters indicate statistically significant differences among groups; groups sharing the same letter are not significantly different. The asterisk (*) indicates statistically significant differences between groups.

When examining the descriptive data, the number of melanophores appeared similar across the three experimental tank colours at the three time points assessed: 5, 12, and 25 dph (Figure 3A). Although melanophores were counted at each dph, aggregation at 5 dph was insufficient, which likely compromised the accuracy of quantification at that time point. Consequently, statistical analyses were only performed for the 12 and 25 dph datasets. Before statistical modelling, an exploratory analysis was performed to evaluate the relationships between the anatomical area where melanophores were quantified (measured area), the number of melanophores, and dph. This analysis revealed two main patterns: (1) a positive correlation between the measured area and the number of melanophores, and (2) a positive correlation between dph and both variables (Figure 3B). Based on these findings, subsequent analyses focused on melanophore per measured area, expressed as the number of melanophores per mm^2^.

Statistical results indicated no significant differences in melanophore per mm^2^ among larvae reared in white (12 dph: 95% CI = 508–606 melanophores/mm^2^; 25 dph: 95% CI = 344–409 melanophores/mm^2^), light-blue (12 dph: 95% CI = 487–582 melanophores/mm^2^; 25 dph: 95% CI = 330–393 melanophores/mm^2^), or grey tanks (12 dph: 95% CI = 489–584 melanophores/mm^2^; 25 dph: 95% CI = 331–394 melanophores/mm^2^) (Figure 3C,D). However, a significant reduction in melanophore per mm^2^ was observed from 12 to 25 dph across all treatments (*p* < 0.0001), suggesting that although both the number of melanophores and the measured area increase with dph, the expansion of the measured area occurs at a faster rate (Figure 3C,D).

### 3.3. Sl Response

**Figure 4:**
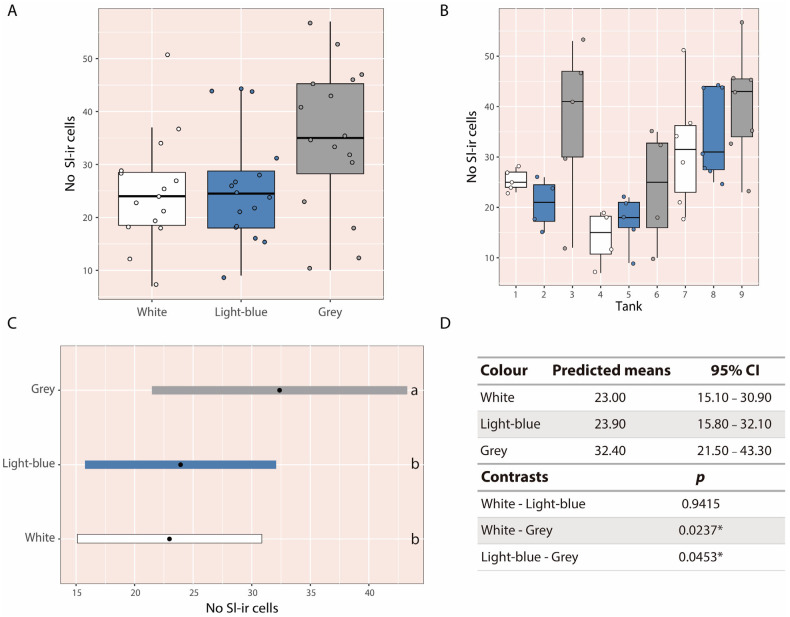
(**A**) Boxplot showing the number of Sl-ir cells in larvae at 60 dph, grouped across all experiments. (**B**) Boxplots for each experimental replicate analyzed individually. (**C**) Adjusted means with 95% confidence intervals for the tank colour factor. (**D**) Predicted means, 95% confidence intervals, and pairwise contrasts among the three tank colours. Different letters indicate statistically significant differences among groups; groups sharing the same letter are not significantly different. The asterisk (*) indicates statistically significant differences between groups.

Larvae reared in grey tanks appeared to show a higher number of Sl-ir cells at 60 dph compared to those reared in white or light-blue tanks based on descriptive comparisons (Figure 4A). This pattern was consistently observed in the descriptive inspection of the pooled data from all three replicates as well as when replicates were plotted separately, with larvae reared in grey tanks exhibiting higher Sl-ir cell counts (Figure 4B). Statistical analysis confirmed these differences: larvae in grey tanks (95% CI: 21.50–43.30) had significantly more Sl-ir cells than those in light-blue (95% CI: 15.80–32.10; *p* = 0.0453) and white tanks (95% CI: 15.10–30.90; *p* = 0.0237) (Figure 4C,D). No significant differences were detected between larvae reared in white and light-blue tanks. Overall, larvae in grey tanks exhibited approximately 36% more Sl-ir cells than those in white tanks and 35% more than those in light-blue tanks.

### 3.4. Somatic Growth

Although total length and body weight were measured at 5, 12, 25, 60, and 90 dph, differences among tank colour treatments were evident only at 90 dph (Figure S1). Consequently, only data from 90 dph were included in the statistical analyses. Neither the interaction between colour and density (*p* = 0.88) nor density as a main effect (*p* = 0.45) were statistically significant. Because adding density did not substantially improve model fit (ΔAIC < 2), the simpler model including tank colour as the only fixed factor was retained.

**Figure 5:**
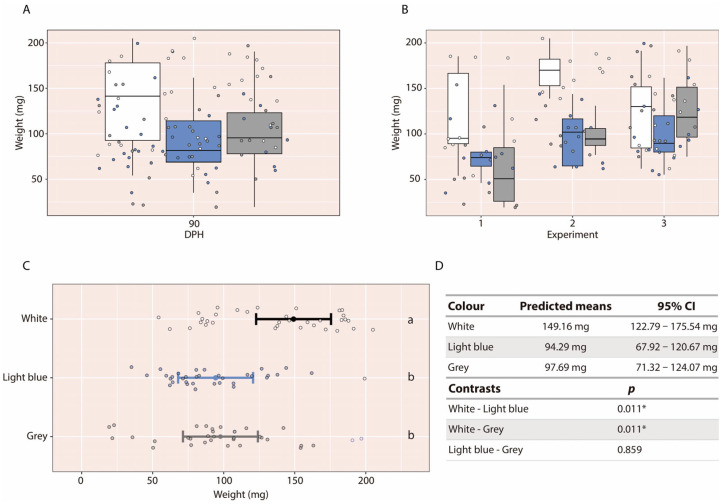
(**A**) Boxplot of larval body weight at 90 dph, grouped across experiments. (**B**) Boxplots for each experimental replicate individually. (**C**) Back-transformed means with 95% confidence intervals for the tank colour factor. (**D**) Predicted means, 95% confidence intervals, and pairwise contrasts among the three tank colours. Note that the white tank is represented by a black line. Different letters indicate statistically significant differences among groups; groups sharing the same letter are not significantly different. The asterisk (*) indicates statistically significant differences between groups.

Larvae reared in white tanks appeared to exhibit greater final body weight than those reared in grey or light-blue tanks based on descriptive comparisons (Figure 5A). To further illustrate this pattern, the 90 dph datasets from each experimental replicate were examined graphically. In all three cases, larvae reared in white tanks consistently showed higher average body weight, regardless of the experiment (Figure 5B). Statistical analyses confirmed this pattern: larvae reared in white tanks (95% CI: 122.79–175.54 mg) had significantly higher final weights than those reared in light-blue tanks (95% CI: 67.92–120.67 mg; *p* = 0.011) and grey tanks (95% CI: 71.32–124.07 mg; *p* = 0.011) (Figure 5C,D). No significant differences in body weight were detected between larvae reared in light-blue and grey tanks.

**Figure 6:**
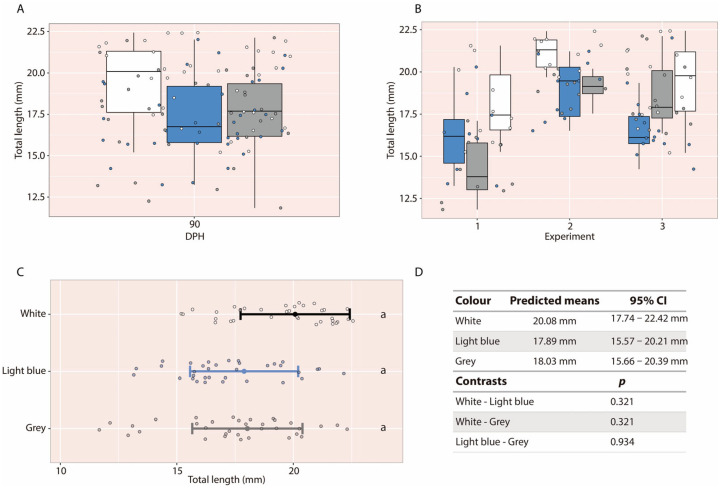
(**A**) Boxplot of larval total length at 90 dph, grouped across experiments. (**B**) Boxplots for each experimental replicate individually. (**C**) Back-transformed means with 95% confidence intervals for the tank colour factor. (**D**) Predicted means, 95% confidence intervals, and pairwise contrasts among the three tank colours. Note that the white tank is represented by a black line. Different letters indicate statistically significant differences among groups; groups sharing the same letter are not significantly different.

In the case of total length, no significant differences were detected among tank colour treatments. Final total length did not differ significantly between larvae reared in white tanks (95% CI: 17.74–22.42 mm), light-blue tanks (95% CI: 15.57–20.21 mm), or grey tanks (95% CI: 15.66–20.39 mm) (Figure 6).

### 3.5. Gh Response

**Figure 7:**
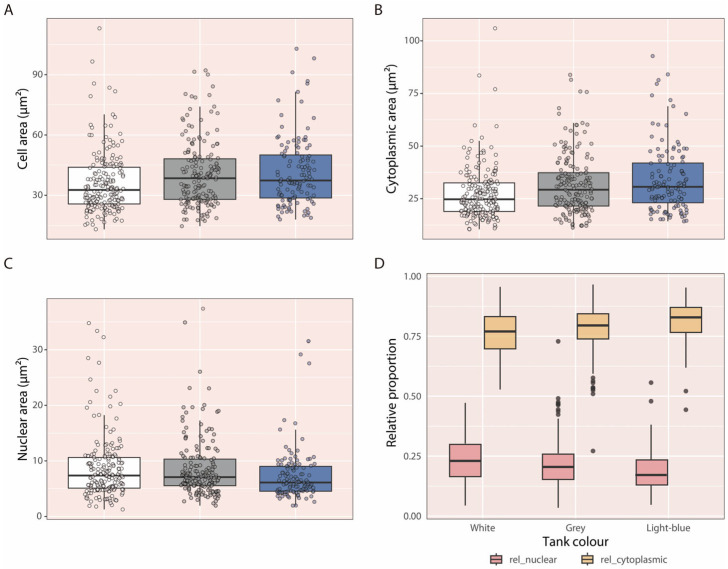
Boxplots of total cell (**A**), cytoplasmic (**B**), and nuclear (**C**) areas of Gh-ir cells from larvae at 90 dph, comparing the three experimental tank colours and pooling all experiments. (**D**) Boxplots showing the relative contribution of nuclear (rel_nuclear) and cytoplasmic (rel_cytoplasmic) areas to total cell area in Gh-ir cells from larvae at 90 dph, across the three tank colours.

At 90 dph, descriptive analyses suggested that Gh-ir cells tended to have larger total and cytoplasmic areas in larvae reared in light-blue and grey tanks, whereas nuclear area tended to be larger in larvae reared in white and grey tanks. On average (±SD), total cell area was 36.30 ± 15.58 µm^2^, 41.63 ± 17.37 µm^2^, and 40.44 ± 16.39 µm^2^ for larvae reared in white, light-blue, and grey tanks, respectively. Cytoplasmic area averaged 27.56 ± 12.79 µm^2^, 34.29 ± 16.11 µm^2^, and 31.79 ± 14.34 µm^2^, while nuclear area was 8.74 ± 5.76 µm^2^, 7.34 ± 4.60 µm^2^, and 8.65 ± 5.38 µm^2^, respectively (Figure 7A–C). This tendency was further supported by plotting the relative proportions of cytoplasmic and nuclear areas relative to total cell area: the cytoplasmic fraction seemed lowest in larvae reared in white tanks, intermediate in grey tanks, and highest in light-blue tanks, whereas the opposite pattern was observed for the nuclear fraction (Figure 7D).

These patterns were consistent across both analyzed areas and among replicates. No interaction between region and tank colour was detected, and the final model included only tank colour as a fixed effect and cell area as a covariate. Cell area was significantly correlated with both response variables. Tank colour had a significant effect on nuclear area (*p* < 0.0001) and marginal effect on cytoplasmic area (*p* = 0.0566).

**Figure 8:**
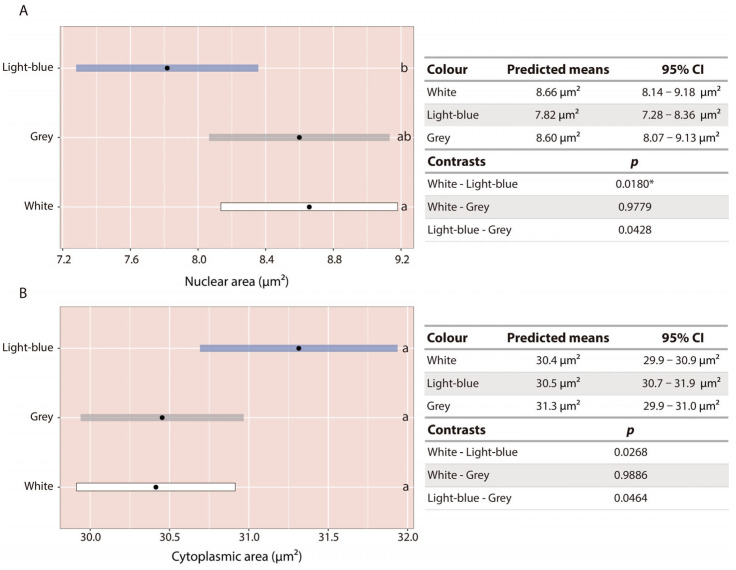
Predicted means with 95% confidence intervals and pairwise comparisons among the three tank colours for (**A**) nuclear and (**B**) cytoplasmic areas of Gh-ir cells. Different letters indicate statistically significant differences among groups; groups sharing the same letter are not significantly different. The asterisk (*) indicates statistically significant differences between groups.

Post hoc contrasts revealed that nuclear area was significantly greater in Gh-ir cells of larvae reared in white tanks (95% CI = 8.14–9.18 µm^2^; *p* = 0.0180) compared to those reared in light-blue tanks (95% CI = 7.28–8.36 µm^2^). Although differences between grey and light-blue tanks did not remain significant after Bonferroni correction (*p* = 0.0428), they followed the same trend (Figure 8A). For cytoplasmic area, statistical significance was lost after correction, but a consistent tendency toward larger values was observed in larvae reared in light-blue tanks (95% CI = 30.7–31.9 µm^2^) compared to white (95% CI = 29.9–30.9 µm^2^; *p* = 0.0268) and grey tanks (95% CI = 29.9–31.0 µm^2^; *p* = 0.0464) (Figure 8B).

### 3.6. Sex Ratio

**Figure 9:**
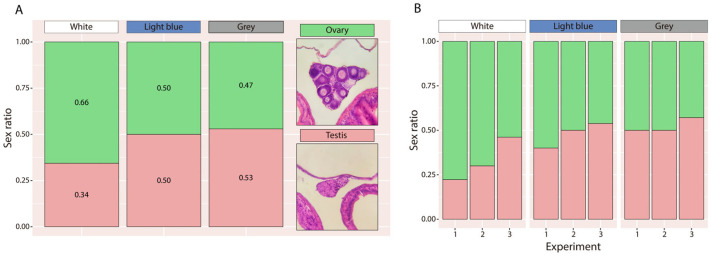
Sex ratio at 90 dph for larvae reared in the three experimental tank colours, presented both grouped (**A**) and by individual replicate (**B**). Representative histological images of ovaries and testes are also shown.

A higher proportion of females was observed in larvae reared in white tanks (34%, IC95% = 20–52%) compared to those reared in light-blue (50%, IC95% = 32–68%) or grey tanks (53%, IC95% = 36–69%) (Figure 9A). However, the higher proportion of females observed in white tanks was not statistically significant. Consistent with other descriptive results, this trend was also apparent when each replicate was examined individually (Figure 9B).

### 3.7. Skeletal Development

**Figure 10:**
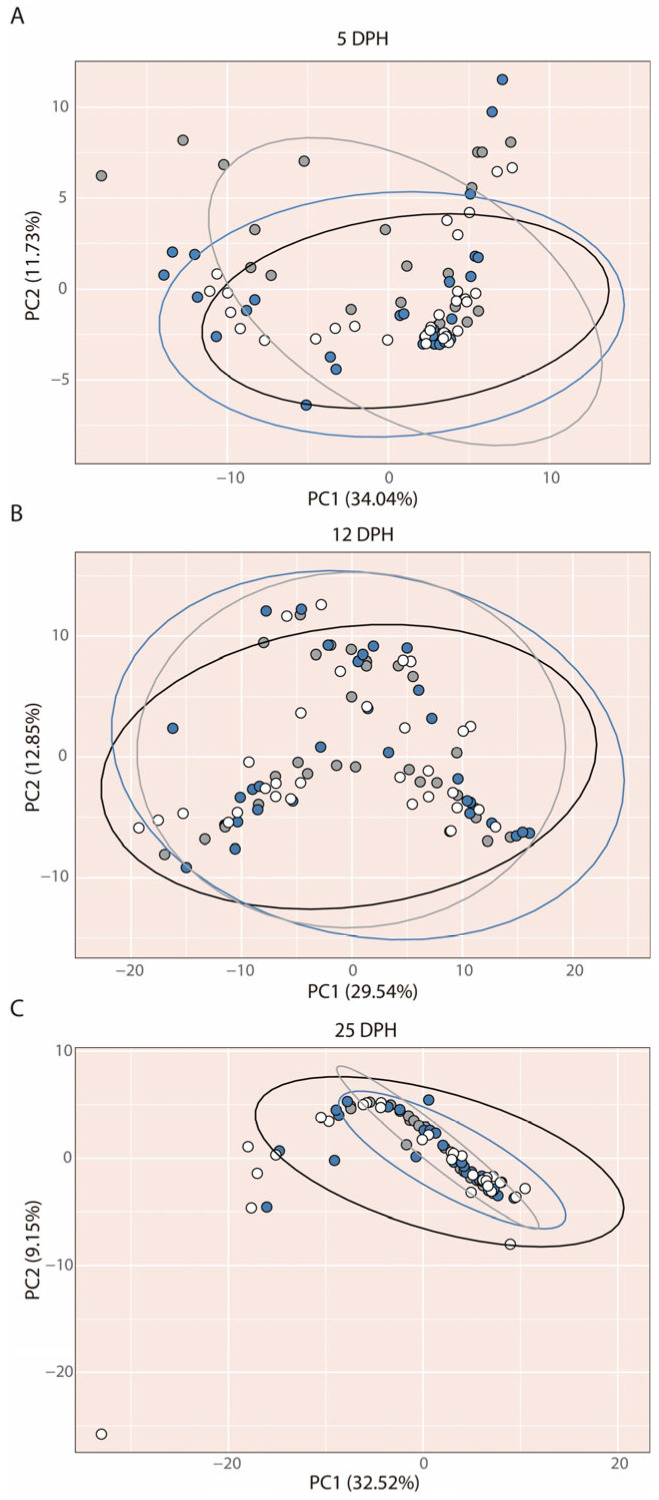
Principal component analysis scatter diagram of *C. dimerus* skeleton structures. The three experimental tank colours are delimited by ellipses. (**A**) 5 dph, (**B**) 12 dph, and (**C**) 25 dph larvae.

The Principal Component Analysis (PCA) revealed no differences in skeletal development among the different tank colours at any of the three sampling days (Figure 10). This result was supported by the PERMANOVA analyses, which detected no significant effect of tank colour (*p* > 0.05). These outcomes were consistent across experimental replicates and skeletal regions.

At 5 dph (Figure 10A), PC1 explained 34.04% of the variance, primarily driven by the onset of ossification in the axial skeleton, particularly in the most rostral abdominal vertebrae and neural arches. PC2 accounted for a smaller proportion (11.73%) and was also linked to axial skeleton development, in this case reflecting the chondrogenesis of the most caudal neural and haemal arches.

At 12 dph (Figure 10B), PC1 represented 29.54% of the variance, with major contributions from neurocranial bones such as the frontal and parietal, as well as the completion of ossification in the most caudal neural and haemal arches. PC2 captured 12.85% of the variance and was associated with the chondrogenesis of the distal radials in the dorsal fin.

At 25 dph (Figure 10C), PC1 explained 32.52% of the variance, mainly influenced by fin-support structures, including the proximal radials of the dorsal and anal fins and the dorsal ribs. Although no effect of tank colour on skeletal development was detected, larvae reared in white tanks displayed a larger PCA ellipse, suggesting greater variability in skeletal development at this stage (Figure 10C). This pattern was not evident at 5 or 12 dph, when ellipse sizes were similar (Figure 10A,B).

## 4. Discussion

In general terms, the present study showed that survival was lower in fish reared in white tanks compared to those reared in light-blue and grey tanks. Conversely, larvae from white tanks reached a higher final body weight, while total length showed a similar, though non-significant, trend. Gh-ir cells exhibited a larger nuclear area in larvae reared in white tanks and a tendency toward larger cytoplasmic areas in those reared in light-blue tanks. The number of melanophores was similar across treatments, but larvae from grey tanks displayed a higher number of Sl-ir cells. Sex ratios tended to be female-biased in white tanks. Skeletal development did not differ among colour treatments and followed the expected chondrogenesis and ossification sequence for the species.

Across different fish species, higher survival rates have often been reported in darker tanks [4,45,46]; in some cases, greater survival has been observed in light-coloured tanks [6], as in the present study, while in others no significant differences were detected [47]. Interestingly, higher survival rates are usually associated with better growth performance, which is not the case here [6,9,45,46,48]. The greater body weight observed in white tanks cannot be attributed to differences in density, as this factor was experimentally controlled. Lower growth rates are often associated with reduced survival in the literature, and this relationship may be linked to stress responses, which are energy-demanding processes that can increase catabolic activity and, consequently, limit growth [6,9,45]. In this study, survival curves showed similar overall patterns among treatments; however, when mortality events occurred, they were consistently more pronounced in larvae reared in white tanks. These mortality events were observed across all tank colour treatments and were associated with general rearing conditions inherent to long-term experiments, which, rather than undermining the results, make them more representative of realistic aquaculture or research settings. Although stress was not directly measured, this pattern suggests that larvae reared against a white background may be more sensitive under these rearing conditions. In this context, higher stress levels in larvae reared in white tanks represent a plausible explanation. In line with this possibility, background colour has been shown to modulate stress physiology in other teleost larvae; for example, in *Amphiprion frenatus*, only larvae reared in white tanks exhibited increased whole-body cortisol levels in response to an acute stress challenge [49]. At the same time, the higher visual contrast between food and a white background has been reported to enhance feed visibility and detection efficiency [6,46], which may contribute to increased food intake and growth. Taken together, our results suggest that white backgrounds may simultaneously enhance feeding efficiency while increasing stress responsiveness, potentially contributing to reduced survival. However, further experimental evidence is required to clarify the mechanisms underlying these patterns. Overall, our findings highlight that greater growth does not necessarily translate into better welfare conditions, as it may occur at the cost of reduced survival.

Regarding skin pigmentation, no differences were observed in the number of head melanophores among tank colours at either of the two developmental stages analyzed. Previous studies in *C. dimerus* reported a greater number of head melanophores from 15 dph onwards in larvae, as well as in adult scales, in fish reared or kept in black tanks compared to those in white tanks [30,35]. This pattern is not restricted to *C. dimerus*, as in several species, fish tend to display lighter skin coloration when reared in lighter tanks and darker pigmentation in darker tanks [4], with only one exception so far, where the opposite trend was observed in *Hippocampus abdominalis* [50]. However, most of these studies compared only black and white backgrounds, leaving a gap in our understanding of how more subtle tank colours influence skin pigmentation [4,8,51,52,53,54,55,56,57]. Kasagi et al. (2020) [58] used a similar colour palette to the one applied in the present study and observed only slight tendencies in *O. mykyss* skin pigmentation changes. Larvae reared in white tanks tended to be brighter in the dorsal region than those from grey tanks, and these, in turn, were brighter than those from light-blue tanks. The use of subtler colours in both studies may explain the absence of significant differences, suggesting that stronger background contrasts might be required to elicit detectable pigmentation responses Another possible explanation is that longer exposure times may be necessary to reveal more marked differences, which seems likely in *C. dimerus*, as in the present study we observed, at 60 dph, a higher number of Sl-ir cells in larvae reared in grey tanks [30,31]. Quantifying head melanophores was not possible at 60 dph due to their exponential increase in number and their distribution in more than one layer, which hindered accurate counting. Therefore, future studies evaluating skin pigmentation in older larvae using alternative methodologies that allow a direct correlation with Sl responses would be of great interest. As mentioned before, larvae reared in white and light-blue tanks showed a smaller number of Sl-ir cells compared to those reared in grey tanks at 60 dph. A smaller number of Sl-ir cells in fish reared or maintained in white tanks is consistent with previous findings in both adults and larvae of *C. dimerus*. In this species, white backgrounds have been associated with fewer Sl-ir cells in larvae, as well as with lower *sl* and *slr* expression levels in adults [30,31,35]. Additionally, studies in the red drum *(Sciaenops ocellatus*) reported significantly higher plasma and pituitary Sl concentrations in fish exposed to black backgrounds than in those maintained under light backgrounds [59]. When evaluating the effects of illumination, plasma Sl concentrations were low during the light phase, significantly increased during the early-dark phase, and declined toward the late-dark phase. In contrast, Sl levels remained low throughout a 24 h constant-light period [57]. Moreover, Sl concentrations were significantly higher in optic-tract–sectioned and enucleated fish than in control fish during both the early-dark and early-light phases of the 24 h light–dark cycle [60]. Together, these results indicate that plasma Sl levels are elevated in the absence of light and under dark background conditions. However, in the present study, illuminance was comparable between white and grey tanks and was lower in light-blue tanks. Therefore, the observed differences in Sl-ir cell number cannot be explained solely by light intensity. Interestingly, Kasagi et al. (2020) [58] reported a similar pattern among light–blue, white, and grey tanks. The fish reared in the light-blue tank showed similar *sl* expression compared to those reared in a white tank. The expression levels of *sl* in fish reared in the grey tanks fell between those of the fish reared in black and white tanks. Taken together, our results support a role of Sl in background adaptation in *C. dimerus*, and highlight that, while previous studies have primarily attributed Sl differences to changes in light intensity (i.e., black–white background contrast), our findings, together with those of Kasagi et al., (2020) [58], indicate that when luminance is comparable among treatments, the spectral composition of the background (chromaticity) may differentially modulate Sl responses. It is also noteworthy that even when illuminance differs among treatments, chromaticity may still play a role in modulating Sl responses. In our study, light-blue tanks showed lower illuminance values than white and grey tanks, yet the Sl pattern observed in this treatment did not resemble that typically reported for fish maintained under dark or black backgrounds. This suggests that the spectral properties of the background, rather than light intensity alone, can differentially influence Sl regulation.

Regarding somatic growth, larvae reared in white tanks reached a higher final body weight, while total length showed a similar, although non-significant, trend. Previous studies on *C. dimerus* larvae reported no differences in length between tank colours up to 30 dph [35], whereas in the present study, growth differences became evident only at 90 dph. Although measurements at 5, 12, 25, and 60 dph were descriptive, differences emerged at later stages, suggesting that long-term exposure may be required for tank colour effects on growth to become apparent. Enhanced growth in white tanks has also been reported for *Carassius auratus* [61], *Perca fluviatilis* [62], *Diplodus sargus* [63], *Paralichthys olivaceus* [47], *Verasper moseri* [64,65], and *Scophthalmus maximus* [66]. In most species showing improved growth under white backgrounds, this effect has been primarily attributed to increased visual contrast between feed and background, leading to higher food intake. Improved feed visibility may act alone or in combination with neuroendocrine mechanisms involving orexigenic peptides. Gh, beyond its central role in promoting somatic growth, has also been linked to appetite regulation, as Gh-overexpressing fish exhibit increased food intake, supporting its orexigenic function [67]. In our study, Gh-ir cells in larvae reared in white tanks exhibited larger nuclear areas than those reared in light-blue tanks, together with a tendency toward smaller cytoplasmic areas in larvae from white and grey tanks. Larger nuclear size may indicate higher transcriptional activity, whereas reduced cytoplasmic area may reflect increased peptide secretion. Together, these Gh-ir cell characteristics are consistent with a more active synthesis and secretion process in larvae reared in white tanks. Consistent with these findings, larvae of *Seriola dumerili* reared in white tanks showed higher mRNA expression levels of several growth-related genes (*igf i*, *igf ii*, *igf bp2*, *igf bp3*, and *igf bp5*) compared to those reared in darker or green backgrounds [68].

Somatolactin is part of a broader genetic and hormonal network regulating skin pigmentation, which also involves Mch and α-melanocyte-stimulating hormone (α-Msh) [69]. While α-Msh promotes melanin dispersion in skin melanophores, Mch induces melanin aggregation and inhibits α-Msh synthesis and secretion [69,70]. In adult *C. dimerus*, anatomical studies have shown that Mch-ir fibres are located in close proximity to Sl-ir cells [33]. In addition to an increase in Sl-ir cell number observed in fish kept in black tanks, a marked rise in α-Msh-ir cell numbers has also been reported, whereas fish reared in white tanks exhibited larger Mch-ir nuclear areas [30]. Moreover, Pérez Sirkin et al. (2012) [34] demonstrated direct contacts between Mch-ir fibres and Gh-ir cells and showed that Mch increases *gh* transcript levels and stimulates Gh release in pituitary cultures. Consistently, *C. dimerus* kept in white tanks presented a greater number of Mch neurons with larger nuclei and higher *mch* transcript levels compared to those reared in black tanks. Juveniles from white tanks also displayed greater body weight and total length, suggesting that Mch may be involved in somatic growth regulation in this species. Evidence for *mch* involvement in growth responses was also reported in *V. moseri*, where larvae reared in white tanks showed higher numbers of Mch-ir cells and elevated *mch* levels in the brain, pituitary, and plasma [64,65,71,72]. Furthermore, Mch has been proposed to play an orexigenic role in several teleosts [73], supporting the idea that enhanced somatic growth may reflect increased food intake modulated by this hormone. However, in *O. mykiss*, although increased *mch* levels were reported in white tanks, this was not accompanied by higher *gh* expression or enhanced somatic growth [58]. Therefore, future studies incorporating direct measurements of Mch, α-Msh, and feed intake will be necessary to further assess the potential contribution of these mechanisms under the experimental conditions used in the present study.

Sex ratio is a key parameter influencing the viability of both aquaculture stocks and wild populations [74,75,76]. This ratio results from the processes of sex determination and differentiation [77]. In gonochoristic species, sex determination can be genotypic (GSD) or environmental (ESD), depending on whether it is controlled by genetic or chromosomal differences or modulated by environmental factors [78]. These mechanisms are now understood as endpoints of a continuum rather than mutually exclusive systems [79,80,81]. In the present study, although no statistically significant differences were observed in sex ratios, a clear female-skewed tendency (66%) was recorded in white tanks compared to grey (47%) and light-blue (50%) tanks, where the ratios were almost or exactly 1:1. To our knowledge, this is the first study evaluating sex ratio responses of *C. dimerus* under different environmental conditions, providing preliminary evidence that this species may exhibit some degree of environmental influence on sex determination. In other fish species, several environmental factors have been shown to influence sexual development, including temperature, density, hypoxia, pH, and photoperiod [82,83,84,85,86,87]. In many cases, male-skewed sex ratios appear to be associated with stress-mediated mechanisms involving corticosteroids such as cortisol. This has been demonstrated in the pejerrey (*O. bonariensis*), where thermal stress during the critical period of sex determination induces cortisol elevation and subsequent masculinization [85,88]. Regarding tank colour, the only study available so far in *Paralichthys lethostigma* reported significantly male-biased sex ratios in blue tanks compared to black and grey ones. Whole-body cortisol levels were higher in fish reared in blue tanks during the sex-determining period, suggesting that stress-induced corticosteroids may play a role in modulating sex determination [10]. Additionally, in *Nematobrycon palmeri*, rearing under different LED light colours also affected sex ratio, showing a significant female bias under blue and red lights (≈3:1) and a tendency towards female bias under white light (≈60:40), consistent with the trend observed in our study [89]. Although the differences observed in *C. dimerus* were not statistically significant, such a shift could still be relevant in aquaculture contexts. Taken together, these results provide a first insight into how tank colour might influence sexual development in *C. dimerus*.

In adult *C. dimerus*, males are generally larger than females [25], indicating sexual size dimorphism at later life stages. However, information on sex-specific growth patterns during larval stages is currently lacking. Therefore, the possibility that female larvae may exhibit larger body size than males during early developmental stages cannot be excluded. In *Dicentrarchus labrax*, sexual size dimorphism has been shown to emerge before histological differentiation of ovaries and testes, with females exhibiting significantly higher growth rates during early development [90,91]. These findings suggest that growth is not necessarily a consequence of sex, but rather that individuals growing faster or reaching larger sizes may have a higher probability of differentiating as females. Consistently, Papadaki et al. (2005) [92] reported that fast-growing populations of *D. labrax* displayed a higher proportion of females. Nevertheless, the relationship between growth and sex determination does not appear to be unidirectional across species. In *Anguilla japonica*, females exhibited slower growth rates than males during the critical period of sex determination, and growth restriction resulted in an increased proportion of females [93]. Together, these studies indicate that early growth trajectories can modulate sex ratios in a species-specific manner. In the present study, in addition to a tendency towards a higher female proportion in fish reared in white tanks, this treatment was also associated with higher final body weight and lower survival. On one hand, these results are compatible with a scenario in which differential growth occurring prior to gonadal differentiation may contribute to ovarian differentiation, as suggested for other teleost species. Alternatively, a differential mortality between sexes cannot be excluded. However, analysis of the survival curves indicates that the main mortality events occurred predominantly before the expected period of gonadal differentiation. In this context, although both hypotheses remain plausible, our results highlight the need for more detailed studies focused on the period immediately preceding sexual differentiation, incorporating individual growth trajectories. Such approaches will be essential to unravel the mechanisms through which early environmental conditions, such as tank background colour, may influence sex ratios in this species.

Many studies have demonstrated the influence of environmental effects on skeletal development, meristic counts, and incidence of deformities, providing solid evidence of skeletal plasticity [94]. Among these factors are water velocity [95,96,97], temperature [98,99,100], rearing density [101,102], culture intensiveness [103], and nutrition [103,104]. However, only one study has reported an effect of tank colour on jaw deformities, associated with increased walling behaviour in red tanks [12]. In the present study, we selected three developmental stages (dph) based on the description of *C. dimerus* skeletogenesis under standard rearing conditions, which evidenced greater changes in cartilage or bony structures [43]. Neither overall skeletal development nor that of specific regions showed differences among tank colours at the days analyzed. Moreover, the timing, sequence, and degree of skeletal element appearance were consistent with the pattern described under standard conditions. Only at 25 dph—the latest stage analyzed—did larvae reared in white tanks exhibit greater heterogeneity in skeletal development, which may be related to a more sensitive ossification process relative to chondrification, and to greater variability in late-forming compared to early-forming elements [97]. Taken together, these findings suggest that skeletal development in *C. dimerus* shows low plasticity in response to tank colour.

## 5. Conclusions

Overall, the results of this study demonstrate that tank colour influences multiple aspects of larval development, mainly affecting survival, body weight, and the endocrine responses of Sl and Gh. These findings highlight the importance of considering tank colour in research and rearing practices, providing evidence of its role as an early environmental cue that can shape physiological development and ultimately influence larval performance.

## Data Availability

The raw data supporting the conclusions of this article will be made available by the authors on request.

## Supplementary Materials

The following supporting information can be downloaded at: https://www.mdpi.com/article/10.3390/ani16030466/s1, Figure S1: Descriptive statistics of body weight and total length of larvae reared in tanks of different colours across sampling days (dph). Values are presented as mean ± SD for each tank colour and sampling point.
