# Supplementary material for: Different Colours, Different Outcomes: Tank Colour Shapes Larval Survival, Growth, and Endocrine Response in Cichlasoma dimerus
Source: Animals (Basel). 2026 Feb 2;16(3):466. doi: 10.3390/ani16030466 (PMC12896578; doi:10.3390/ani16030466)
Supplement: Supplementary file 1 [file animals-16-00466-s001.zip › animals-4091901-supplementary.pdf]

DPH	Colour	Weight (mean ± SD)	Total length (meand ± SD)
5	White	2.59 ± 0.56 mg	5.28 ± 0.34 mm
	Light-blue	3.13 ± 0.99 mg	5.38 ± 0.38 mm
	Grey	2.35 ± 0.53 mg	5.35 ± 0.24 mm
12	White	6.80 ± 2.21 mg	7.12 ± 0.80 mm
	Light-blue	7.08 ± 1.99 mg	7.55 ± 0.87 mm
	Grey	6.95 ± 1.84 mg	7.37 ± 0.64 mm
25	White	19.27 ± 7.90 mg	10.06 ± 2.00 mm
	Light-blue	19.16 ± 3.75 mg	9.84 ± 1.23 mm
	Grey	19.44 ± 6.35 mg	10.16 ± 1.48 mm
60	White	60.14 ± 26.21 mg	14.57 ± 1.86 mm
	Light-blue	64.01 ± 31.44 mg	15.00 ± 2.16 mm
	Grey	60.43 ± 29.16 mg	10.16 ± 1.48 mm
90	White	135.43 ± 42.75 mg	19.52 ± 2.23 mm
	Light-blue	93.14 ± 35.29 mg	17.26 ± 2.25 mm
	Grey	99.57 ± 43.51 mg	17.50 ± 2.69 mm

Figure S1. Descriptive statistics of body weight and total length of larvae reared in tanks of different colours across sampling days (dph). Values are presented as mean ± SD for each tank colour and sampling point.
